# Patterns, Causes, and Mortality Trends of Road Traffic Accidents in Hamedan Province, Iran: A Spatiotemporal Epidemiological Analysis (2011–2024)

**DOI:** 10.34172/jrhs.11510

**Published:** 2025-10-18

**Authors:** Ebrahim Jalili, Salman Khazaei, Mahdi Khazaei, Sanaz Omidi

**Affiliations:** ^1^Department of Emergency Medicine, School of Medicine, Hamadan University of Medical Sciences, Hamadan, Iran; ^2^Department of Epidemiology, School of Public Health, Hamadan University of Medical Sciences, Hamadan, Iran; ^3^Research Center for Health Sciences, Institute of Health Sciences and Technologies, Hamadan University of Medical Sciences, Hamadan, Iran; ^4^Department of Nursing and Midwifery, School of Nursing and Midwifery, Hamadan University of Medical Sciences, Hamadan, Iran; ^5^Student Research Committee, Hamadan University of Medical Sciences, Hamadan, Iran

**Keywords:** Road traffic accidents, Mortality, K-prototype clustering, Hamadan province, Time series forecasting

## Abstract

**Background:**

Road traffic accidents (RTAs) are a major cause of death, especially in developing countries. This study analyzed RTA patterns and trends in Hamadan province, Iran, from 2011 to 2024.

**Study Design:**

A cross-sectional study.

**Methods:**

Data on 6,488 road traffic fatalities, excluding non-road transport deaths, were obtained from the Forensic Medicine Organization. Demographics, injury type, location, and mode of transport were analyzed. Then, spatial clustering was performed using the K-prototype algorithm, with cluster quality assessed via the Silhouette Score. Finally, mortality trends were forecasted using the Prophet model with 95% prediction intervals and evaluated using RMSE, MAE, MASE, and MAPE.

**Results:**

The mean age of victims was 49 ± 21 years, with most deaths observed in the 15–44 age group. Most victims were male (75%), urban residents (60%), married (69%), and had not completed secondary education (41%). Fatalities mainly occurred at the scene (48%) or en route to the hospital (43%), with head/face injuries and hemorrhage as the leading causes. Spatial analysis revealed three clusters, with Famenin having the highest mortality (73.7 per 100,000). Mortality was projected to decline from 18.27 (16.01, 20.55) in 2025 to 9.57 (7.37, 11.69) in 2028, rising slightly to 13.94 (11.83, 16.17) in 2029.

**Conclusion:**

Overall, the findings emphasize the need for targeted regional interventions (e.g., road safety education, enhanced emergency services, and infrastructure upgrades) to reduce RTA mortality in the high-risk areas of Hamedan Province.

## Background

 Road traffic accidents (RTAs) are one of the leading causes of death, physical injuries, and socio-economic damages worldwide. With the growing population and uneven development of road transportation, the need to establish safe and sustainable infrastructure has become increasingly urgent. According to the World Health Organization, nearly 1.19 million people died due to road accidents and over 135 million people were injured in 2021; these incidents annually account for 3%–5% of countries’ gross domestic product.^1^ Developing countries suffer the highest burden, with over 90% of fatalities, mainly due to weak infrastructure, low public awareness, and poor compliance with traffic laws.^2-4^ The main factors contributing to accidents include inadequate road design, poor traffic management, human error, and negligence.^5^ Based on the reports of the World Bank, developing countries own only 30% of the world’s vehicles, accounting for 74% of road accidents.^6^

 Iran ranks 113th among 175 countries, with a mortality rate of 20 deaths per 100,000 population. Overall, 84,213 people died from road accidents in Iran between 2013 and 2019.^1,7^ Studies show that pedestrians, motorcyclists, and vehicles are the primary groups involved, with most accidents occurring on roads and during busy summer days.^8,9^ The burden of road traffic injuries includes years of life lost, disability, and treatment costs, imposing significant economic pressure on society.^9-13^

 The temporal and spatial analyses of accidents can help identify dense accident-prone areas as well as locations with high-risk potential, playing a key role in traffic planning, emergency response, and rescue operations. Different techniques, such as kernel density estimation and spatial statistical indices (e.g., Getis-Ord Gi and Moran’s I), have been employed to analyze cluster patterns and the spatial distribution of accidents.^14-16^ For instance, Jackson and Sharif used the Gi index to analyze the spatial clusters of rain-related accidents in Texas, USA.^17^ Similarly, Samia applied comparable methods to identify hazard-prone areas on the Haraz road.^9^ Furthermore, investigating the relationship between human factors, weather conditions, and environmental hazards with accident locations, especially in high-density areas, can provide a suitable basis for designing educational, preventive, and infrastructural strategies. Other researchers, such as Perrels et al in Finland^7^ and Yang et al in China,^18^ have highlighted the role of environmental conditions (e.g., weather and landslide risk) in increasing road accidents, emphasizing the necessity of considering these factors in road safety planning.

 Official statistics indicate that Hamadan province has faced serious challenges regarding road accidents in recent years. According to reports, 439 people lost their lives in traffic incidents in this province in 2024. These figures demonstrate a worrying state of road safety in the region and underscore the urgent need for preventive measures and improvements in transportation infrastructure. Therefore, this research aims to examine the spatial pattern of road accident density across the counties of Hamadan province using spatiotemporal analyses. Awareness of the spatial distribution of high-accident areas can aid in making decisions related to transportation and urban traffic planning at the regional scale, identifying accident-prone zones, and developing and implementing technical, legal, and preventive measures to enhance road safety.

## Methods

###  Study design and setting 

 This cross-sectional study was conducted after obtaining approval from the Ethics Committee of Hamadan University of Medical Sciences. The research completely adhered to ethical guidelines, ensuring the confidentiality of personal information.

 Data collection in forensic medicine starts at the scene of the incident, where investigators meticulously record and collect evidence to avoid contamination. This entails documenting the incident’s circumstances and keeping samples safe in containers that are marked and impossible to tamper with. Then, physical and biological samples are sent to forensic laboratories for in-depth examination, which includes imaging, toxicological and clinical testing, and autopsy procedures, to ascertain the cause and circumstances of death. Throughout this process, a rigorous chain of custody is upheld to guarantee the evidence’s integrity and admissibility in court. Eventually, all gathered data and conclusions are methodically entered into databases for additional examination and reporting to the appropriate legal and policy-making bodies.

 The dataset included 6,488 traffic accident fatalities in Hamadan province from 2011 to 2024, obtained from the Forensic Medicine Organization. Deaths from non-road transport (rail, air, or water) were excluded from the investigation. All records were verified as unique, with the cause of death, year, and county of occurrence recorded for each case. Missing data were minimal, affecting only a few non-critical variables, but did not influence the main analyses. Moreover, data preprocessing ensured accuracy and transparency across all analyses. Traffic accident mortality was analyzed using a combination of clustering, spatial analysis, and time series forecasting, providing a comprehensive assessment of both spatial patterns and temporal trends.

###  K-prototype clustering algorithm

 K-means is a widely used unsupervised clustering algorithm that partitions data into K distinct clusters by assigning each data point to the nearest centroid, typically based on Euclidean distance. The algorithm iteratively updates cluster centroids by calculating the mean of all points within each cluster to minimize the sum of squared distances between points and their assigned centroids. However, K-means is inherently limited to numerical data.^19,20^ On the other hand, the K-prototype is designed to handle datasets that include both numerical and categorical variables, overcoming the restriction of K-means, which only works with numerical data. This prototype adapts the clustering process by combining distance calculations for numeric features with a measure of dissimilarity for categorical features, making it suitable for real-world datasets with mixed data types.^21^ The K-prototype algorithm allows the use of both numeric and categorical data.^22^ Assume a set *n* objects 𝑋 = {𝑋_1_, 𝑋_2_, ⋯, 𝑋_n_}, 𝑋_𝑖 _= {𝑋_𝑖1_, 𝑋𝑖_2_, ⋯, 𝑋𝑖𝑚} consists of 𝑚 attributes (𝑚_𝑟_ is numerical attributes, 𝑚_𝑐_ is categorical attributes, 𝑚 = 𝑚_𝑟 _+ 𝑚_𝑐_). The goal of clustering is to partition *n* objects into k disjoint clusters 𝐶 = {𝐶_1_, 𝐶_2_, ⋯, 𝐶𝑘}, where 𝐶_𝑖_ is the i-th cluster center. The distance 𝑑 (𝑋_𝑖_, 𝐶𝑗) between 𝑋_𝑖_ and 𝐶_𝑗_ can be calculated as follows:


(1) $$d\;(X_{i},𝐶j)=d_{r}\;(X_{i},𝐶j)+\gamma \;dc\;(X_{i},𝐶j)$$

whereas *d*_r_ (X_i_, C_j_) represents the distance between the numerical attributes, and *d*_c_(X_i_, C_j_) denotes the dissimilarity between the categorical attributes. In addition, *γ* is a weighting parameter that balances the contribution of categorical features in the clustering process.


(2) $$d_{r\left(x_{i},c_{j}\right)}=\sum_{l=1}^{P}{\left|x_{il}-c_{jl}\right|}^{2}$$

(3) $$d_{c\left(x_{i},c_{j}\right)}=\sum_{l=p+1}^{m}\delta \left(x_{il},c_{jl}\right)$$

(4) $$\delta \left(x_{il},c_{jl}\right)=\left\{\begin{array}{l} 0,\;when\;x_{il}=c_{jl}\\ 0,\;when\;x_{il}\neq c_{jl} \end{array}\right.$$

In Eq. (2), 𝑑_𝑟_ (𝑋_𝑖_, 𝐶𝑗) is the squared Euclidean distance measure between cluster centers and an object on the numerical attributes. Further, 𝑑_𝑐_ (𝑋_𝑖_, 𝐶𝑗) represents the simple matching dissimilarity measure on the categorical attributes, where 𝛿 (𝑥_𝑖𝑙_, 𝑐𝑗𝑙) = 0 for 𝑥_𝑖𝑙_ = 𝑐_𝑗𝑙_ and 𝛿 (𝑥_𝑖𝑙_, 𝑐𝑗𝑙) = 1 for 𝑥_𝑖𝑙_≠𝑐_𝑗𝑙_. 𝑥_𝑖𝑙_ and 𝑐_𝑗𝑙_, 1 ≤ 𝑙 ≤ 𝑝 are values of numerical attributes. However, 𝑥_𝑖𝑙_ and 𝑐_𝑗𝑙_, 𝑝 + 1 ≤ 𝑙 ≤ 𝑚 are values of categorical attributes for object *i*. Furthermore, the cluster center *j. *𝑝 denotes the number of numerical attributes, and 𝑚−𝑝 is the number of categorical attributes.

 The Elbow method was applied to determine the optimal number of clusters. This method evaluates the within-cluster sum of squares for various values of K.^23^ In this study, a significant drop in within-cluster sum of squares was observed at K = 3, indicating the optimal number of clusters.

###  K-prototype clustering algorithm and feature normalization

 Several features were considered for the K-prototype analysis, including county-level latitude and longitude, number of fatalities, road classification, and year. Predominant road type refers to the road category (within-city, outside-city, or dirt/rural) that constitutes the majority of the road network in each county. Mortality rates per 100,000 population were calculated solely for visualization purposes (represented as point sizes in scatter plots) but not for clustering. To account for differences in scale among numeric variables (latitude and longitude), minimum-maximum normalization was applied before clustering, ensuring robust clustering and allowing for the reliable identification of three distinct high-risk clusters in Hamadan province. The scatter plot visualization also annotated counties with mortality rates above 15 per 100,000 population, providing a clear visual representation of high-risk areas and supporting the spatial interpretation of clustering results.

 Several prior studies have classified traffic accident data based on geographic and temporal factors.^24-26^ However, this research specifically focused on clustering high-risk accident zones within Hamadan province based on year, location, number of fatalities, and road classification. This multi-criteria approach allows for a more detailed and actionable understanding of spatial risk distribution.

###  Heatmap analysis

 Heatmaps were generated using kernel density estimation to visualize the spatial distribution of traffic accidents. Kernel density estimation calculates the density of events in a geographic area, which is then represented by a color gradient map. These heatmaps provide a visual representation of areas with higher accident concentrations.^27^

###  Time series forecasting with prophet

 Prophet, also known as FbProphet, is an open-source forecasting tool developed by the Core Data Science team at Facebook.^27^ It was designed to model time series data using a decomposable structure^28^:

 y(t) = g(t) + s(t) + h(t) + e(t)

 where g(t) is the trend component (linear or logistic), and s(t) denotes the seasonality component modeled via a Fourier series (daily, weekly, or yearly). In addition, h(t) and e(t) are irregular holiday effects and error terms, respectively.

 In this study, the Prophet model was applied to forecast annual traffic accident mortality rates in Hamadan province. Annual mortality data were used as model inputs, and yearly seasonality was incorporated. No separate training-validation split was applied since there was a limited number of annual observations. Moreover, model performance was evaluated using multiple standard accuracy metrics, including root mean square error (RMSE), mean absolute error (MAE), mean absolute percentage error (MAPE), and mean absolute scaled error (MASE). These metrics generally provide insight into the average magnitude of prediction errors, their proportional deviation from observed values, and the model’s performance relative to a simple naive baseline. Forecasted mortality rates for counties with smaller populations, such as Famenin, exhibited wider 95% confidence intervals, reflecting greater uncertainty associated with smaller sample sizes. The transparent reporting of these intervals allows for a more accurate assessment of spatial risk and annual mortality trends.

###  Tools and libraries

 All analyses were conducted using the Python programming language via libraries such as Pandas and NumPy for data handling and preprocessing, and Matplotlib and Seaborn for data visualization. The other libraries included Scikit-learn and Kproto for implementing the K-prototype clustering algorithm, Prophet for time series forecasting, and Seaborn heatmap function for constructing spatial heatmaps.

## Results

###  Demographic and basic characteristics of fatalities

 A total of 6,488 traffic accident fatalities in Hamadan province from 2011 to 2024 were analyzed, with a mean age of 49.06 ± 20.93 years (range 1–94). The highest mortality occurred in the 15–30 (1,178 cases; 24.3%) and 30–44 (1,168 cases; 24.1%) age groups. At the same time, a considerable number of deaths were observed in older age groups, with 1,047 cases (16.1%) in the 45–59 age group and 999 cases (15.4%) among individuals aged ≥ 60 years. This age distribution shifted the overall mean upward, while the majority of fatalities remained concentrated in the 15–44 age group. Most victims were male (75.0%; 3,718), residents of urban areas (60.1%; 2,982), married (69.0%; 1,527), and had less than a high school diploma (41.4%; 2,683). Fatalities peaked in 2011 (9.8%; 637) and 2012 (9.0%; 581), while they were lowest in 2023 (5.9%; 384) and 2024 (5.9%; 381). The analysis of the place of death revealed that 48.1% of victims died at the scene of the accident (2,379), while 42.8% died en route to the hospital (2,117). Of all fatalities, 42.3% (2,745) were drivers.

###  Injury patterns and associations

 Chi-square analysis confirmed a significant association between injured body region and victim status (χ^2^ = 67.09, df = 18, *P* < 0.001). Based on the findings, head and face injuries were most common across drivers (47.1%), passengers (45.6%), and pedestrians (46.7%). Pedestrians also had higher arm/hand (44.7%) and leg (39.9%) injuries than drivers. The results demonstrated that injuries to the pelvis and posterior torso were relatively rare (6.2% and 1.6%, respectively).

 A significant relationship was found between patient transport method and cause of death (χ^2^ = 66.04, df = 24, *P* = 0.000). Hemorrhage was the leading cause, especially in patients transported by ambulance (45.5%) and private vehicles (62.9%). Moreover, head trauma was common, particularly in patients transported by police vehicles (73.3%). Deaths from multiple fractures were more frequent with other transport methods (8.1%), possibly due to delays or inadequate equipment.

 Furthermore, the transport method was significantly associated with injury type (χ^2^ = 30.89, df = 18, *P* = 0.0296), with over 90% of patients transported by ambulance regardless of injury type. Private vehicles and police cars accounted for less than 7% and 1% respectively. Additionally, the place of death was linked to transport method (χ^2^ = 37.71, df = 12, *P* = 0.0002) and time of accident (χ^2^ = 50.32, df = 12, *P* < 0.0001), indicating variability in transport use and accident timing by location.

###  Mortality by road type

**Figure 1:**
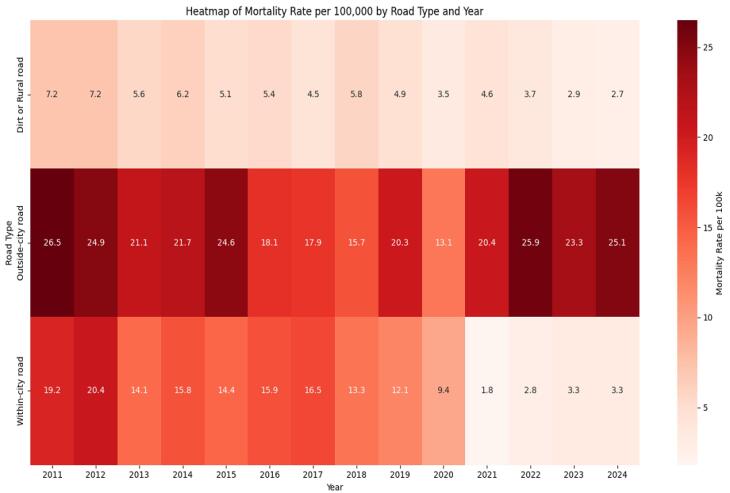
Mortality rates by road type in Hamadan province (2011–2024)

 Based on the road type variable, the highest mortality rates were reported on outside city roads, with rates of 26.5 and 25.9 deaths per 100,000 population recorded in 2011 and 2022, respectively. Further, the years 2012, 2015, and 2024 showed some of the highest mortality rates on these roads, with rates of 24.9, 24.6, and 25.1 deaths per 100,000 population, respectively. Within city roads had the highest mortality rates in 2012 and 2011, with rates of 20.4 and 19.2 deaths per 100,000 population, respectively. The lowest mortality rates throughout the study period (2011–2024) were observed on dirt and rural roads (Figure 1). Ultimately, outside city roads were identified as hotspots for traffic mortality and were considered the most hazardous road type in terms of fatality occurrence.

###  Clustering analysis

 The Elbow method was used to determine the optimal number of clusters (k) by examining the reduction in the sum of squared errors across different k values. Based on the results (Table 1), the decrease in the sum of squared errors noticeably slowed after k = 3, representing a suitable balance between model complexity and clustering quality. Therefore, k = 3 was selected as the optimal number of clusters.

**Table 1:** Sum of squared error values for clusters ranging from K = 1 to K = 9, highlighting the elbow point at K = 3

**Number of clusters (K)**	**Sum of squared errors**
1	286.25
2	70.10
3	23.83
4	10.92
5	5.80
6	2.44
7	1.09
8	0.50
9	0.30

 To ensure fair contribution of all numerical features in clustering, latitude, longitude, and mortality rate were normalizedprior to applying the K-prototype algorithm. To evaluate clustering quality, the Silhouette Score was calculated using only numerical features, resulting in a score of 0.570. This indicates that the clusters were reasonably well-separated and internally cohesive. K-prototypesclustering identified three distinct clusters among the nine counties of Hamadan province: Cluster 0 (red): Kabudarahang, Bahar, Asadabad, Razan, Nahavand, and Tuyserkan, Cluster 1 (blue): Malayer and Hamadan, and Cluster 2 (green): Famenin.

 counties in Cluster 0 are located in areas with average latitude and longitude. Cluster 1 comprises counties with lower latitude, while Cluster 2 includes counties with higher latitude and longitude. The distribution of road types across clusters is mixed, reflecting diversity in infrastructure and traffic conditions. This cluster assignment was kept fixed for all subsequent analyses to ensure consistent spatial interpretation and reliable visualization of high-risk areas.

 Based on the examination of the clusters, counties with higher mortality rates were grouped together, while variations in road types helped interpret spatial patterns. Nonetheless, the clustering algorithm primarily relied on numerical variables. This approach provides a clear visualization of high-risk areas and highlights potential associations between geographic location, road characteristics, and mortality rates, which can inform targeted interventions and policy planning.

**Figure 2:**
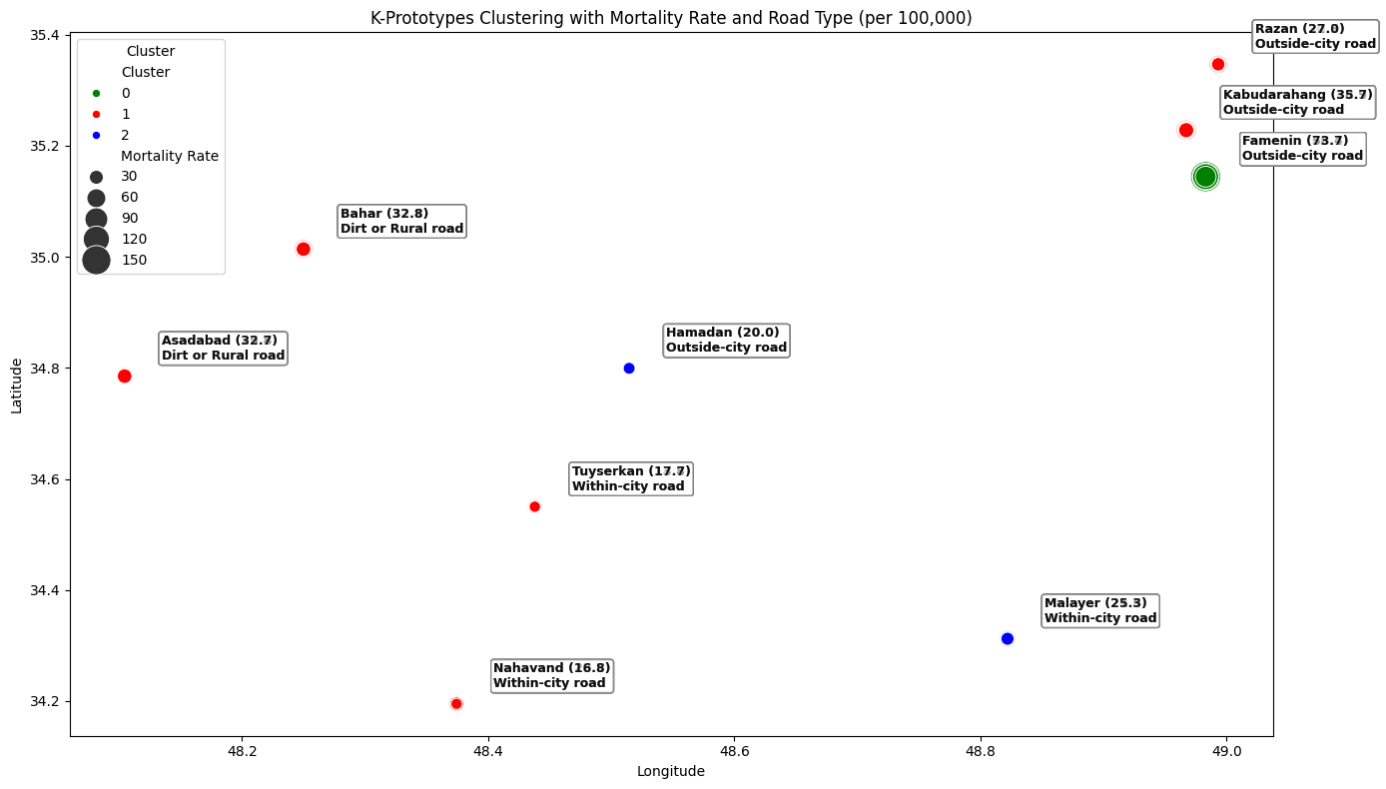
K-Prototype clustering of mortality rate by county in Hamadan province

Figure 2 presents the distribution of traffic accident mortality rates (per 100,000 population) across the nine counties, stratified by road type. Based on empirical evaluation, Cluster 0 (red) and Cluster 1 (blue) had mortality rates ranging from 16.8 to 35.7 and from 16.8 to 32.7, respectively. Moreover, Cluster 2 (green) included Famenin with the highest observed rate of 73.7 per 100,000 population. The spatial distribution highlights clear geographic and infrastructural disparities across the province, influenced by both fatality rates and road environment characteristics.

###  Spatial heatmap analysis

**Figure 3:**
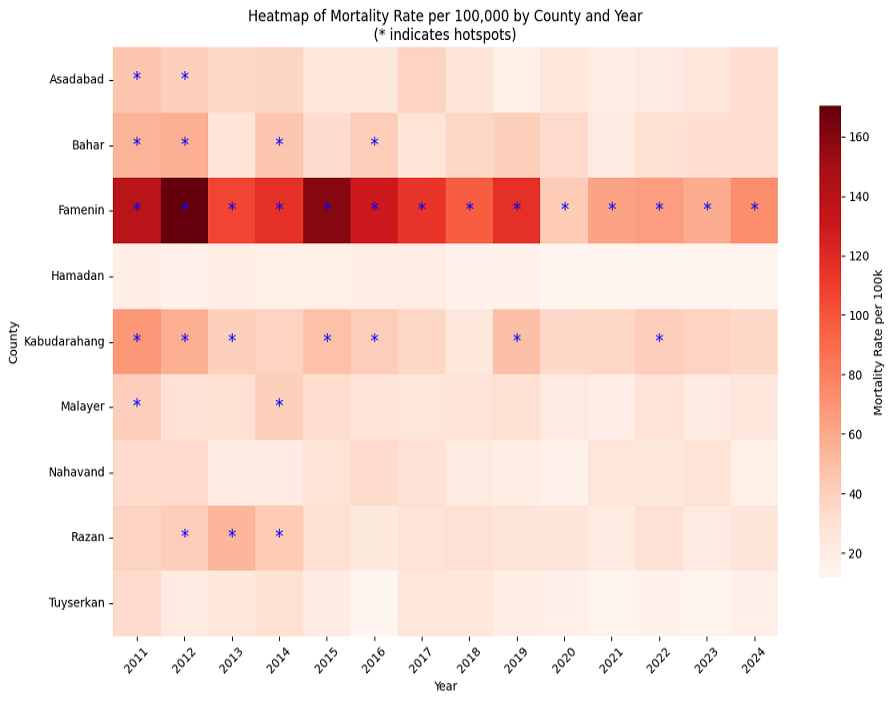
Heatmap of mortality rate by county in Hamadan province

 Based on the results of the heatmap analysis, a red color spectrum was employed to depict the density and severity of traffic accident mortality across the counties of Hamadan province. In this map, darker shades of red indicate higher concentrations of fatalities, representing so-called hotspots with elevated mortality rates, while lighter shades correspond to areas with lower risk. This visual representation facilitates clearer identification of the geographical clusters of traffic-related issues and critical zones. The gradual fading of red intensity in certain regions over the years reflects a relative decrease in traffic mortality rates, which may be attributed to improvements in road infrastructure, increased public awareness, or enhanced emergency medical services. The highest population-adjusted mortality hotspots over the 13-year study period were observed in Famenin, Kabudarahang, and Bahar counties. Specifically, Famenin county exhibited peak annual mortality rates in 2012 and 2015, highlighted on the map by very dark red hues and star symbols. Such concentrated high mortality in these areas could be due to various factors, including poor road quality, lack of warning signs, excessive driving speeds, or long distances to emergency healthcare facilities. Conversely, Hamadan, Nahavand, and Tuyserkan counties were identified as low-risk areas, depicted with lighter shades of red on the map (Figure 3). From an emergency medicine and public health perspective, identifying these hotspots is critical, as it enables targeted allocation of healthcare resources and preventive measures to reduce further fatalities. Improving access to emergency services, implementing focused traffic safety education, and enforcing stricter regulations in these regions can contribute to lowering mortality rates. Furthermore, spatial analyses (e.g., heatmaps) provide valuable tools for monitoring temporal trends and evaluating the effectiveness of intervention programs. Ultimately, the presented heatmap not only identifies high-risk areas but also plays a vital role in strategic planning aimed at reducing traffic accident mortality and enhancing road safety (Figure 3).

###  Time series forecasting

 To forecast traffic-related mortality rates, the Prophet time series model was applied at both the provincial and county levels, incorporating yearly seasonality. On the other hand, no separate training-validation split was performed due to the limited number of annual observations. Model performance was evaluated using four standard metrics, providing complementary insights into prediction accuracy (RMSE = 1.73, MAE = 1.45, MAPE = 5.85%, and MASE = 0.66). These values indicate good model fit, with predictions deviating on average by 5.85% from observed values and outperforming a simple baseline model (MASE < 1).

 Forecasted results demonstrated a declining trend in mortality rates in Hamadan province, from 18.27 (16.01, 20.55) per 100,000 in 2025 to 9.57 (7.37, 11.69) in 2028, followed by a slight increase to 13.94 (11.83, 16.17) in 2029. Counties with smaller populations (e.g., Famenin) showed wider 95% confidence intervals, reflecting higher uncertainty.

**Figure 4:**
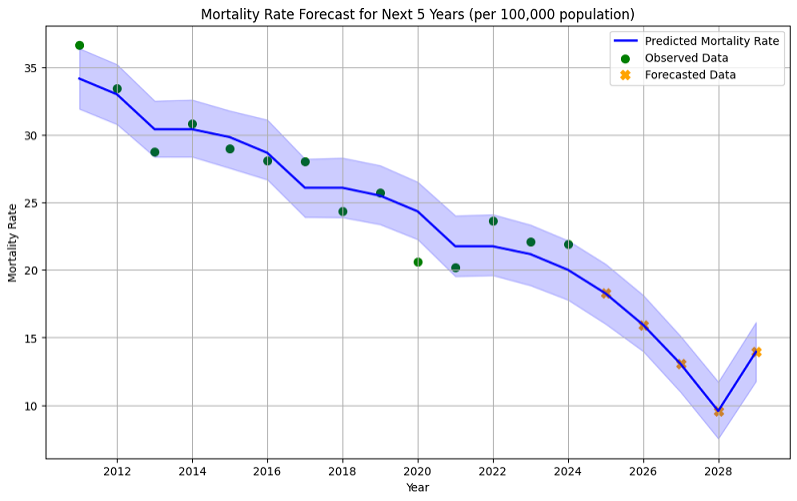
Historical and forecasted trend of mortality rate in Hamadan province (2011–2029)

Table 2 presents projected mortality rates for each county from 2025 to 2029 with 95% confidence intervals. For instance, the mortality rate of Hamadan county is expected to decline from 12.47 (10.74, 14.37) in 2025 to 10.61 (8.82, 12.51) in 2029, while Famenin county is projected to decrease from 46.78 (18.99, 75.09) to 16.22 (11.03, 44.08). Wider intervals in smaller counties highlight the need for cautious interpretation of predictions. Overall, these forecasts indicate a generally declining trend in traffic-related mortality across the province, although counties with persistently higher rates (e.g., Famenin and Kabudarahang) may require targeted public health interventions (Figure 4).

**Table 2:** Predicted traffic mortality rates per 100,000 population by county in Hamadan province (2025–2029)

**County**	**MR**_2025_ ** (95% CI)**	**MR**_2026_ ** (95% CI)**	**MR**_2027_ ** (95% CI)**	**MR**_2028_ ** (95% CI)**	**MR**_2029_ ** (95% CI)**
Hamadan	12.47 (10.74, 14.37)	12.01 (3.71, 10.13)	11.54 (9.79, 13.33)	11.07 (9.28, 12.85)	10.61 (8.82, 12.51)
Malayer	20.96 (15.29, 25.59)	19.83 (14.82, 24.85)	18.72 (13.69, 24.18)	17.59 (12.80, 22.38)	16.48(11.22, 21.33)
Nahavand	20.15 (12.74, 27.04)	19.39 (13.32, 25.89)	18.63 (11.76, 25.21)	17.86 (11.29, 24.91)	17.09 (9.75, 24.20)
Tuyserkan	12.32 (6.42, 18.12)	11.14 (5.35, 17.19)	9.97 (3.98, 15.72)	8.79 (2.92,14.56)	7.61 (1.86, 13.86)
Razan	19.89 (11.76, 27.59)	18.29 (10.97, 26.28)	16.69 (9.97, 23.83)	15.09 (7.63, 22.50)	13.49 (5.11, 21.04)
Kabudarahang	30.92 (19.73, 41.19)	29.39 (18.22, 40.25)	27.86 (17.70, 38.39)	26.32 (16.27, 27.83)	24.78 (13.20, 36.12)
Asadabad	20.22 (11.34, 28.26)	18.93 (10.60, 27.28)	17.65 (9.63, 26.27)	16.37 (8.03, 24.89)	15.08 (6.70, 22.76)
Famenin	46.78 (18.99, 75.09)	39.15 (14.38, 66.89)	31.51 (4.04, 58.97)	23.51 (1.81, 51.57)	16.22(11.03, 44.08)
Bahar	25.69 (15.82, 35.42)	24.19 (14.79, 33.65)	22.67 (12.61, 31.78)	21.16 (11.44, 30.88)	19.64 (8.74, 30.01)
Total	18.27 (16.01, 20.55)	15.94 (13.69, 18.11)	13.04 (10.83, 15.22)	9.57 (7.37, 11.69)	13.94 (11.83, 16.17)

*Note*. MR: Mortality rate; CI: Confidence interval.

 These forecasts emphasize encouraging trends, suggesting that ongoing prevention and intervention strategies may be effective in reducing traffic-related fatalities across the province. However, counties with persistently higher predicted rates (e.g., Famenin and Kabudarahang) should be prioritized for targeted public health initiatives and enhanced emergency response capabilities.

## Discussion

 This study explored the patterns and underlying causes of traffic-related fatalities in Hamadan province over a 13-year span. An analysis of 6,488 recorded deaths due to traffic accidents was conducted, offering a comprehensive overview of mortality trends and influencing factors. The average age of the deceased was 49.06 years. Notably, a higher death rate was observed among individuals aged 15–44, underscoring the significant impact of traffic incidents on the younger and economically active population of the province. In addition, men were disproportionately represented in road traffic fatalities compared to women, a pattern which is consistent with the results of previous research. This disparity is likely influenced by societal norms in the studied populations, where men tend to participate more frequently in outdoor or high-risk activities.^29,30^ However, Jiwane et al reported that individuals aged 41–50 were the most affected group,^31^ while Marak et al found the highest impact among those aged 51–60.^32^ These discrepancies could be attributed to demographic variations across the studied populations. Regarding demographic factors, there are significant variations in the characteristics of motor vehicle accidents across different countries and regions within developing nations. Young people, particularly adolescents and young adults, are disproportionately affected by traffic injuries, a trend well-documented in some studies.^33,34^ Given that many individuals in this age range are at the peak of their economic productivity, RTAs have imposed substantial economic burdens on affected countries.^35-37^

 One of the key findings of the study is that the highest frequency of injuries occurred in the head and facial areas, highlighting the critical importance of preventing head injuries in traffic accidents. Head and brain injuries were the main causes of death and disability, emphasizing the need for urgent medical attention and preventive measures (e.g., seat belts and helmets).^38^ Different factors, such as increased vehicle numbers, poor road conditions, low usage of seatbelts and helmets, and weak enforcement of traffic laws, have exacerbated this issue.^39,40^ In contrast, Obioha et al identified thoracic and abdominal injuries as the second and third leading causes of death.^30^ Meanwhile, Chourasia et al reported hemorrhage as the second most frequent cause of death.^41^ Likewise, Sete and Alemu found that polytrauma and hemorrhage were the second and third most frequent causes of death, respectively.^42^ Mortality due to hemorrhage, especially among those transported by ambulance or private vehicles, underlines the critical need for rapid bleeding control at the accident scene and during transfer to medical centers. This underscores the importance of advanced emergency systems, proper staff training, and suitable equipment to quickly control bleeding and support severely injured patients. Numerous studies have indicated that the “scoop and run” approach improves the survival rates of victims with severe injuries. Any postponement in transferring these patients to specialized healthcare centers needing immediate attention decreases their chances of survival, given the crucial importance of prompt medical intervention.^43^ Our findings revealed that the majority of deaths occurred at the pre-hospital stage, which aligns with the findings of the study performed by Sete and Alemu in Ethiopia.^42^ However, Chourasia et al concluded that most fatalities occur within 12–24 hours after hospital admission.^41^

 In line with the findings of our study,the results of other studies have shown that the highest mortality rate is among drivers, followed by pedestrians who have the second highest share among the victims.^44,45^

 Our results revealed that road conditions significantly affected mortality rates, particularly in rural and suburban roads, which are identified as high-risk areas. These findings conform to previous research outcomes.^44,46^ Several factors contribute to the increased fatality rates on these roads, including inadequate safety measures—especially on unpaved and rural roads—high vehicle speeds in suburban areas, poor geometric design (e.g., sharp bends and winding mountain routes), absence of roadside barriers, lack of proper parking or shoulders, and insufficient lighting along these routes.^46,47^

 The highest mortality rate during the study period was recorded in 2011, with 36.51 deaths per 100,000 people, while the lowest rate was observed in 2024, at 21.9 per 100,000. Based on the observed results and the five-year forecast (2025–2029), the traffic-related mortality rate in Hamadan province has generally shown a declining trend over the 13-year study period, although some fluctuations are evident. While significant reductions in mortality were recorded in certain years, the slight increase in 2029 reflects variability in the overall trend, underscoring the need for closer monitoring and the implementation of more effective interventions to stabilize the downward trajectory. The findings of this study are consistent with those of Shahbazi et al.^44^. This implies that the impact of existing policies and programs on further reducing mortality has diminished despite the effectiveness of the measures taken to reduce road traffic deaths. Therefore, policymakers should pay special attention to designing new and updated interventions for the prevention and management of traffic-related fatalities.

 Additionally, to reduce the mortality rate from RTAs, emergency medical services can be enhanced through technical upgrades, operational improvements, and better equipment. These improvements include increasing the number of ambulances, expanding air ambulance facilities, developing motorcycle emergency services, establishing emergency medical service stations, and reducing the average response time for emergency care.

 This study had several limitations that should be acknowledged. First, although the forensic data provided comprehensive information on traffic accident fatalities, it lacked detailed contextual variables (e.g., weather conditions, driver behavior, and enforcement of traffic regulations), which are known to influence accident risk. Second, the reliance on reported fatalities may lead to the underestimation of the true burden, as non-fatal injuries and unrecorded deaths occurring post-incident were not captured. It should be noted that reliance on forensic databases may have resulted in the underreporting or misclassification of traffic-related fatalities, which could underestimate the true burden of RTA deaths. Third, spatial analysis was performed at the county level, which may obscure intra-county variations and smaller-scale hotspot patterns. Additionally, detailed data on non-urban “country roads” or secondary intercity roads were not available, limiting specific analysis for these road types. The Prophet model provided robust forecasting rather than incorporating exogenous factors (e.g., changes in infrastructure, legislation, or socio-economic trends) that could significantly influence future accident rates. Forecasts used population-adjusted mortality rates, and the accuracy of the Prophet model was limited by the short annual series (13–14 points). Eventually, our data did not include coronavirus disease 2019 or related policy interventions, thereby limiting the analysis to available information.

**Highlights**

- A significant number of fatalities happened either on-site or en route to medical centers.
- Traumatic head injuries and severe bleeding were the most common causes of death.
- The majority of victims were men, particularly those aged between 15 and 44.
- Famenin county recorded the highest rate of road traffic fatalities in the province.
- Forecasts indicated a decline in road traffic deaths from 2025 to 2028, followed by a slight increase in 2029.

## Conclusion

 Rural roads and specific counties, including Famenin, Kabudarahang, and Bahar, were identified as critical high-risk zones in Hamadan province. The majority of traffic fatalities occurred among young males with lower educational levels. Forecasts demonstrated a continued decline in mortality from 2025 to 2028, with a modest rise in 2029. These results highlighted the need for targeted, evidence-based interventions, encompassing traffic safety programs, infrastructure improvements, and enhanced emergency response, in order to effectively reduce road traffic deaths and address spatial disparities in high-risk areas.

## Acknowledgements

 This study was financially supported and approved by the Deputy of Research and Technology at Hamadan University of Medical Sciences. We sincerely thank the Forensic Medicine Organization of Hamadan province for providing access to the traffic accident mortality data. We are also grateful to the Research and Technology Department for their institutional support and approval of the project.

## Competing Interests

 The authors declare no conflict of interests in this study.

## Ethical Approval

 This study was reviewed and approved by the Ethics Committee of Hamadan University of Medical Sciences (Ethical code: IR.UMSHA.REC.1404.377). All data were anonymized prior to analysis, and no personal identifiers were used at any stage. In addition, the study protocol complied with the principles of the Declaration of Helsinki and the guidelines for research involving human data.

## Funding

 This study was financially supported by Hamadan University of Medical Sciences, Hamadan, Iran.
